# Decentralised training for medical students: Towards a South African consensus

**DOI:** 10.4102/phcfm.v9i1.1449

**Published:** 2017-09-28

**Authors:** Marietjie R. de Villiers, Julia Blitz, Ian Couper, Athol Kent, Kalavani Moodley, Zohray Talib, Susan van Schalkwyk, Taryn Young

**Affiliations:** 1 Division of Family Medicine and Primary Care, Faculty of Medicine and Health Sciences, Stellenbosch University, South Africa; 2 Ukwanda Centre for Rural Health, Faculty of Medicine and Health Sciences, Stellenbosch University, South Africa; 3 Department of Obstetrics & Gynaecology, University of Cape Town, South Africa; 4 Departments of Medicine and Health Policy and Management Medical School, George Washington University, South Africa; 5 Centre for Health Professions Education, Faculty of Medicine and Health Sciences, Stellenbosch University, South Africa; 6 Centre for Evidence-based Health Care, Faculty of Medicine and Health Sciences, Stellenbosch University, South Africa

## Abstract

**Introduction:**

Health professions training institutions are challenged to produce greater numbers of graduates who are more relevantly trained to provide quality healthcare. Decentralised training offers opportunities to address these quantity, quality and relevance factors. We wanted to draw together existing expertise in decentralised training for the benefit of all health professionals to develop a model for decentralised training for health professions students.

**Method:**

An expert panel workshop was held in October 2015 initiating a process to develop a model for decentralised training in South Africa. Presentations on the status quo in decentralised training at all nine medical schools in South Africa were made and 33 delegates engaged in discussing potential models for decentralised training.

**Results:**

Five factors were found to be crucial for the success of decentralised training, namely the availability of information and communication technology, longitudinal continuous rotations, a focus on primary care, the alignment of medical schools’ mission with decentralised training and responsiveness to student needs.

**Conclusion:**

The workshop concluded that training institutions should continue to work together towards formulating decentralised training models and that the involvement of all health professions should be ensured. A tripartite approach between the universities, the Department of Health and the relevant local communities is important in decentralised training. Lastly, curricula should place more emphasis on how students learn rather than how they are taught.

## Background

There have been repeated calls for the broadening and enhancing of health workers’ instruction worldwide. These have come from groups and individuals^1,2^ who see a need for health profession educationalists and their institutions to address the quantity, quality and relevance of their graduates.^3^

In South Africa, training institutions are being asked to increase the number of students they graduate, particularly in medicine and nursing. One of the implications of accepting and educating more students is the challenge of finding clinical teaching platforms beyond the traditional tertiary hospital complexes which are already stretched in terms of capacity. The international literature is growing regarding the value of creating *expanded opportunities* at every level at which care is provided.^4,5^

Opportunities for training away from the tertiary academic institutions have been explored in the last two decades with significant growth in the establishment of decentralised training sites. This growth is driven by the desire to broaden the spectrum of student training through exposure to rural and underserved settings.^6,7,8,9^ There is evidence that students trained in rural settings are more likely to consider working in these areas once they qualify than those not exposed to such experiences.^10,11,12^ Decentralised training may assist in addressing the current urban and rural workforce mal-distribution.

Stellenbosch University Collaborative Capacity Enhancement through Engagement with Districts (SUCCEED) aims to improve the quality of HIV/AIDS and related services through capacity development and technical assistance in decentralised learning, operational research, and quality improvement and data. As a sub-component of the project, we undertook to explore the potential of a decentralised training approach nationally because of the complex challenges facing training institutions in establishing such sites.^13,14,15,16,17^ There are no ideal local African models that can serve as an example; so, we engaged in a consultative process working towards a model that could guide a consensus process.

This article describes the first step that consisted of a 2-day workshop to develop a consensus model initially focusing on medical students. It was recognised that much work is being done in decentralised training by other health professions.

Our intention was to create a model for decentralised health professions’ training that can offer context-relevant guidelines for South Africa. This workshop report outlines the key findings from the workshop on which such a model can be based.

## Methods

### Aims and objectives

The aim of the workshop was to initiate a process to develop an overarching model for decentralised undergraduate training for medical students in South Africa. It was thought that a set of principles would be useful to guide and support the establishment and growth of the training facilities. The objective was to discuss and record existing practices with a view to disseminating ideas and agreeing on best practices that could be of mutual benefit.

### Participants

A workshop was held on 8th and 9th October 2015 in Cape Town on decentralised undergraduate medical training. There were 33 participants comprising faculty members from all 9 medical schools in the country, representatives from the National, Eastern Cape and Western Cape Departments of Health and an international expert on community-based education.

### Definitions

For the purposes of the workshop, *decentralised training* was defined as training activities for undergraduate medical students that take place away from tertiary academic complexes – for example: health care centres, primary care clinics and district and rural hospitals.

Other terms in the literature that are used when describing decentralised training are ‘distributed learning’, ‘community-based education’, ‘community-engaged education’, ‘off-campus training’ and ‘rural training’.

### Pre-workshop activities

#### Scoping review

As part of the process of building an evidence-based model for national decentralised training, SUCCEED carried out a scoping review of the international literature. The review explored what decentralised models exist for the training of undergraduate medical students globally, how these models have been implemented and what results have been published.

### Ethical considerations

Ethics approval was obtained from the Stellenbosch University Human Research Ethics Committee (approval number #N16/03/034).

## Results

### Workshop process and findings

Presentation of existing activities: All nine medical schools presented how they were engaged in decentralised training activities. There were wide variations in the duration of decentralised exposure (from short rotations to year-long allocations) as well as considerable diversity in the educational methods with the following elements used: information communication technology (ICT), inter-professional training, community outreach and research. A common enabling feature was a strong connection with the local Department of Health at the district or provincial level.

Some schools reported evaluation results which had resulted in changes to the interaction with students or the academic centre. The challenges included accommodation, transportation and access to ICT. Several schools described the need for more staff development.

Scoping review: The preliminary findings from the scoping review were presented. Five themes emerged that represented essential components of a decentralised training site. They were given as follows:

- responsive student curriculum and assessment
- transformative student experience
- enabling training environment
- effective leadership oversight
- community engagement.

Group work: Participants were split into groups to further discuss the five essential components of decentralised training identified by the scoping review. Groups were asked to establish the key factors to consider within each theme. These are summarised in Table 1.

**TABLE 1:** Key factors for decentralised training.

Components	Key factors
Responsive student curriculum and assessment	Based on burden of disease
	Fit for purpose, socially accountable
	Longitudinal, integrated
	Primary healthcare focused, relevant procedures and clinical skills
	Critical thinking, ethics, law, professionalism
	National and local health system needs
	Service learning and blended learning
Transformative student experience	Continuity of care
	Role definition for all stakeholders
	Community immersion, inter-professional teams, wider exposure
	Student support – briefing, debriefing, mentors, safety
	Logistics and operational needs – learning space, accommodation, etc.
Enabling training environment	Clinical educators trained in appropriate educational theory and skills
	Adequate infrastructure, equipment, space, security
	Appropriate patient mix, practitioner profile, community, quality of care
	Optimal numbers, student-to-trainer ratio, number of practitioners
Community engagement	Training at all levels (clinic, mobile clinic, ward health, community)
	Longitudinal continuous experience
	Attachment to community and households
	Community-oriented primary care
	Engagement of community in governance
	Engagement of students as teachers, peer learning
	Conducting of situational analysis, mapping, assessments
Effective leadership and oversight	Dedicated leadership and oversight, identified champions
	Mission alignment, health facility becomes learning organisation
	Site selection, supervision requirements, costing
	Core curriculum to standardise
	Students to learn in busy clinical settings
	Develop community of practice with everyone, from leadership down
	Demonstrate students’ contribution

**FIGURE 1:**
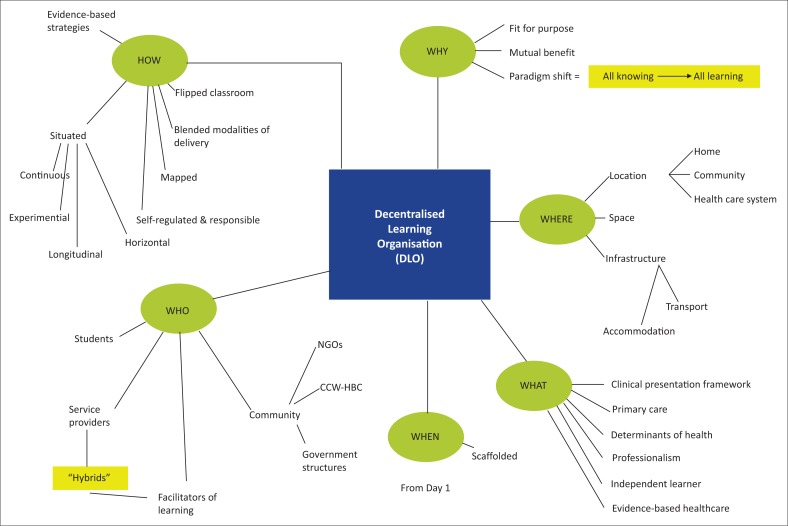
A mind map of decentralised training. This mind map organises a decentralised learning organisation (DLO) in terms of the how, why, where, what, when and who. Under each of these headings, various critical elements that need to be considered are listed. The term ‘hybrids’ refers to clinician educators at the decentralised sites who are also involved in supervising and training students.

**FIGURE 2:**
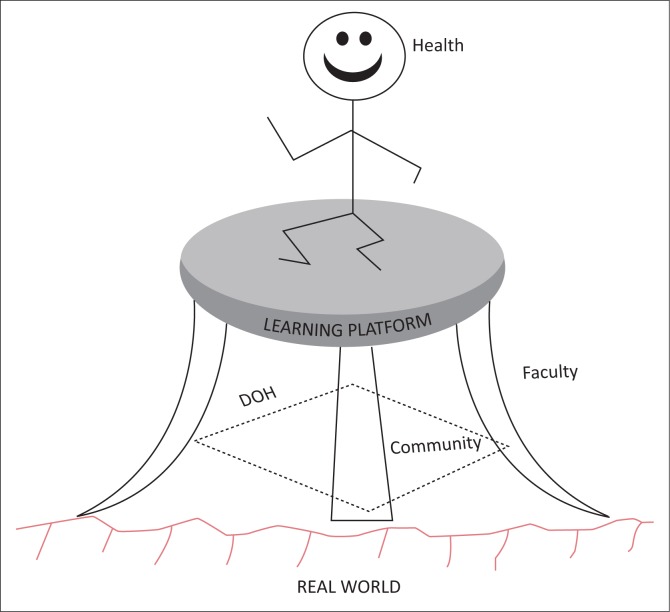
Three-legged stool. This figure uses a traditional African three-legged stool to show that three categories of stakeholders, namely the Department of Health, the community and the academic faculty, are all required to provide a stable decentralised learning platform for health professions students. This interdependent partnership has the power to achieve that despite the uneven and irregular terrain of real-world healthcare. Through supporting learning, such a platform has the potential to improve the health of the population.

**FIGURE 3:**
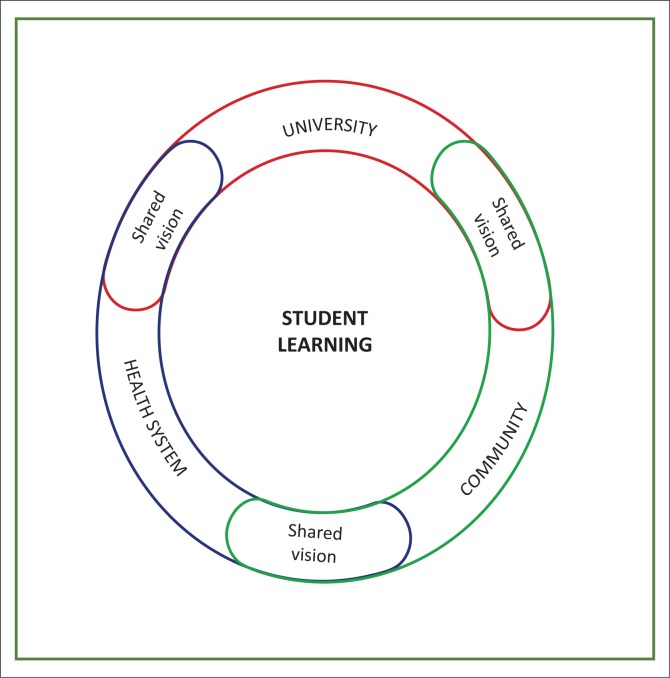
Concentric circles. This diagram depicts decentralised learning as a holistic concept. Student learning is in the centre of two concentric circles. This vision when shared amongst the community, the university and the health system can deliver successful and relevant decentralised learning.

**FIGURE 4:**
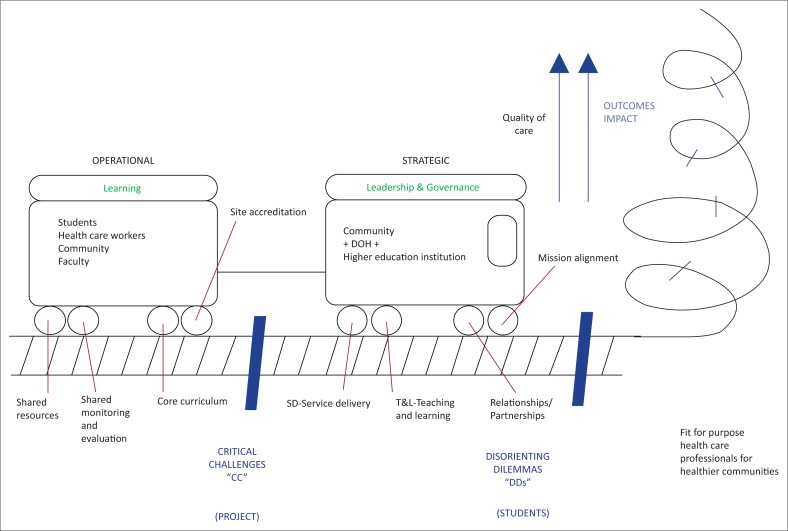
The train model. Decentralised medical education is depicted as a train moving towards the goal of producing fit-for-purpose healthcare professionals for healthier communities, climbing ever higher towards outcomes that will impact on the quality of healthcare and on access to care. The project requires an engine of strategic partnerships with leadership and governance shared amongst higher education institutions, the Department of Health and the communities that are served. This engine will function optimally in the presence of mission alignment, service delivery, teaching and learning and partnerships. It will then be able to pull along the carriage of operational processes required by students, healthcare workers, and community and faculty members. This would be eased by sharing resources, monitoring and evaluation, curriculum and criteria for site accreditation. There are bumps along the line that need to be negotiated. These may be critical challenges within the learning environment, which need to be resolved, or disorienting dilemmas faced by students in the process of learning.

**FIGURE 5:**
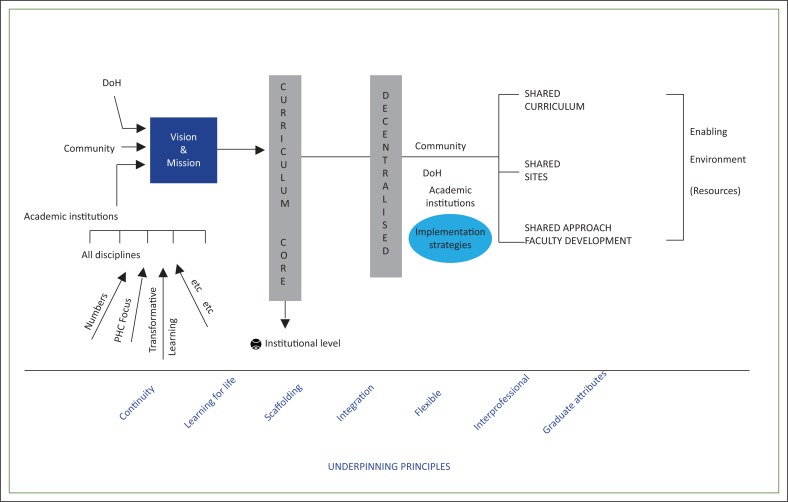
A process model. This model for decentralised learning is characterised by a focus on the process to be followed towards the creation of an enabling decentralised training environment. The model recognises the inputs of all the key role players – the Department of Health, the community in which the decentralised site is established and the academic institution responsible for the training programme. The academic institution’s approach is influenced by its specific context, culture and philosophy. These combine to form a vision and a mission for the learning that takes place at the HPCSA accredited decentralised site, which requires the development first of the core curriculum and then the decentralised site. Implementation strategies require shared resources, namely curricula, sites and opportunities for faculty development. Importantly, this entire process is underpinned by principles that include continuity, life-long learning, approaches to learning, inter-professional education and the embedding of graduate attributes.

Visual depictions: After identifying the key factors, each group was asked to draw an image of decentralised training that arose from their discussions and which would clarify their concept. Figures 1–5 show the images developed by the groups. The figures illustrate the factors necessary in establishing decentralised training and highlight the critical roles in decentralised training sites of three key stakeholders: the community, the Department of Health and the academic institutions.

### Prioritisation of key factors

The workshop participants voted for the key factors identified during the group work to prioritise the elements required to establish and support a decentralised training site. The top five priorities were as follows:

- The availability of ICT: This was seen as essential for mobile learning, data recording, sharing of experiences and to prevent social isolation.
- Longitudinal, continuous rotations: These rotations are proven to be more effective for achieving socially accountable, transformative and clinical learning outcomes.
- Focus on primary care: Primary care emphasis in the curriculum would allow a decentralised rotation to be viewed as an advantage from the student perspective.
- Alignment of the medical school’s mission with a decentralised focus: Each medical school should place emphasis on students acquiring learning outcomes that are best achieved outside of the tertiary academic hospital and which is clearly articulated in the faculty’s mission.
- Responsiveness to student needs: As students of various levels of expertise are allocated to decentralised training, identifying and responding to a variety of student needs is required. Staff at decentralised sites need to be made aware of the range of students’ experience.

## Discussion

Developing models to establish or strengthen decentralised training sites is timely as there is pressure to train greater numbers of medical students and provide a broader base to existing curricula. The SUCCEED workshop provided a representative dialogue focused on developing such a model for establishing decentralised training sites for health professions’ education.

Both the range of participants and the depth of the discussions created a sense of community and momentum around this common area of interest.

We believe there is the potential for developing a national consensus on models for decentralised training in South Africa. Such a process would naturally involve other health professions as many of these have been more active in community-based training than in medicine. There is a need for the development of practical guidelines for any approach to the implementation of decentralised training in health science faculties. The work developed at the workshop was a first step in this process.

SUCCEED was asked to continue the national dialogue and include other key stakeholders such as the National Department of Health, the Health Professions Council of South Africa (HPCSA), the Committee of Medical Deans and the South African Committee of Health Science Deans.

As part of implementing decentralised training, ways of supporting faculty and supervisors need to be developed. It was considered important to reassure staff at decentralised sites that student allocations have not been found to be a hindrance to the efficient and effective functioning of healthcare delivery. Other important factors are ensuring good training sites, understanding the cost of an *ideal facility* and identifying quality and quantity metrics to effectively evaluate decentralised training.

### Workshop recommendations

The following recommendations were synthesised from the workshop proceedings after being reviewed and verified by the participants:

1. There needs to be a change of emphasis from how students are *taught* to how students *learn* throughout their medical school careers. Decentralised training could be a driver in addressing this perceived mal-alignment.
2. It is important to explore the way in which students are deployed and the activities that they carry out during decentralised training. It needs to be established how students can contribute to the service, the enhancement of care and improve academic standards thereby adding value during their decentralised training. This would help in reframing the interaction between medical schools and decentralised health facilities towards a more symbiotic relationship.^18^
3. As part of the collaboration between faculties and health facilities, there is a need to investigate how data collected by the health service can be used to monitor what students are doing and so evaluate their contribution.
4. It would be wise to consider curricular standardisation across the country, especially in terms of decentralised training. This could allow for the development of common tools for monitoring and evaluation, quality assurance and assessment. It would also facilitate the development of common core competencies for medical students in relation to decentralised care to help address the needs of our communities.

## Conclusions

The aim of this workshop was to initiate a process to develop an overarching model for decentralised training in the health professions, discussing current practices with a view to disseminate ideas and agree on best practices. It is our hope that this report will be a catalyst for further discussion and debate. All nine South African medical schools are engaged in decentralised training in some way. The extent of their commitment to this was evident during the workshop. This workshop was a first step in the journey to reaching national consensus on how to achieve such transformation.
